# Early-life origin of prostate cancer through deregulation of miR-206 networks in maternally malnourished offspring rats

**DOI:** 10.1038/s41598-023-46068-1

**Published:** 2023-10-31

**Authors:** Luiz M. F. Portela, Flavia B. Constantino, Ana C. L. Camargo, Sérgio A. A. Santos, Ketlin T. Colombelli, Matheus N. Fioretto, Luísa A. Barata, Erick J. R. Silva, Wellerson R. Scarano, Sergio L. Felisbino, Carlos S. Moreno, Luis A. Justulin

**Affiliations:** 1 Department of Structural and Functional Biology, Institute of Biosciences, Sao Paulo State University (UNESP), Botucatu, SP 18618-689 Brazil; 2 Department of Biophysics and Pharmacology, Institute of Biosciences, Unesp, Botucatu, Brazil; 3 Department of Pathology and Laboratory Medicine, Emory University School of Medicine, Atlanta, GA USA; 4 Department of Biomedical Informatics, Emory University School of Medicine, Atlanta, GA USA; 5 Cancer Signaling and Epigenetics Program, Fox Chase Cancer Center, Philadelphia, PA 19111 USA

**Keywords:** Endocrine system and metabolic diseases, Intrauterine growth

## Abstract

The Developmental Origins of Health and Disease (DOHaD) concept has provided the framework to assess how early life experiences can shape health and disease throughout the life course. While maternal malnutrition has been proposed as a risk factor for the developmental programming of prostate cancer (PCa), the molecular mechanisms remain poorly understood. Using RNA-seq data, we demonstrated deregulation of miR-206-Plasminogen (PLG) network in the ventral prostate (VP) of young maternally malnourished offspring. RT-qPCR confirmed the deregulation of the miR-206-PLG network in the VP of young and old offspring rats. Considering the key role of estrogenic signaling pathways in prostate carcinogenesis, in vitro miRNA mimic studies also revealed a negative correlation between miR-206 and estrogen receptor α (ESR1) expression in PNT2 cells. Together, we demonstrate that early life estrogenization associated with the deregulation of miR-206 networks can contribute to the developmental origins of PCa in maternally malnourished offspring. Understanding the molecular mechanisms by which early life malnutrition affects offspring health can encourage the adoption of a governmental policy for the prevention of non-communicable chronic diseases related to the DOHaD concept.

## Introduction

Non-communicable chronic diseases (NCDs) are reaching epidemic proportions worldwide, with 41 million deaths each year. The most deadly NCDs are related to cardiovascular diseases, followed by several types of cancers, respiratory tract diseases, and diabetes. Almost 80% of these deaths occur in low- and middle-income countries^1^. Although NCDs are diagnosed mainly in adults, a growing body of evidence strongly supports the hypothesis that exposure to adverse conditions during the formative periods disrupts normal developmental biology and predisposes individuals to high-risk NCDs across the lifespan. The reprogramming of physiologic intrauterine/early postnatal development forms the basis of the Developmental Origins of Health and Disease (DOHaD) concept^2^.

The DOHaD concept has emerged over the past 50 years from the outstanding epidemiologic observations of Barker and Osmond (1986) that poor nutrition early in life was associated with increased infant mortality and ischemic heart disease in adults in England and Wales^3^. Nowadays, a broad range of epidemiological and experimental studies have confirmed and extended Barker's observations, consolidating gene-environment interactions during early life as a critical risk factor for the increased incidence of NCDs later in life. Although the initial focus of DOHaD studies has been directed to obesity, diabetes, and cardiovascular disease, exposures to environmental stressors have been considered an important risk factor for the developmental origins of several malignancies, including breast and prostate cancers^4–7^. The first author to propose the prenatal origin of prostate cancer (PCa) was William Gardner in 1995, who states that “*The origins of prostatic diseases, including carcinoma, are to be found in the *in utero* influences upon the developing prostate*”^5^. Later, Keinan-Boker et al. (2009) demonstrated an increased risk of PCa in Jewish men exposed early in life to famine and stress during the Holocaust. Although tragic, this event allowed researchers to explore the potential association between early-life exposure to stress conditions and increased risk for PCa with aging^8^.

In the last decade, clinical and experimental studies have provided significant insights into the molecular mechanisms involved in the developmental origins of PCa. In this regard, intrauterine and early postnatal exposure to estrogens or estrogenic compounds, including endocrine-disrupting chemicals (such as bisphenols and phthalates), have been proven to interfere with prostate developmental biology and the susceptibility of prostatic diseases with aging in both humans^4,9,10^ and rodent models^11–13^. Although the prostate is dependent on testicular androgens for both development and the maintenance of its functional integrity, estrogens also act on prostate gland development and homeostasis and changes in physiologic levels have been associated with the etiology of prostatic diseases. Estrogenic effects on prostate cells are mediated through estrogen receptors alpha (ERα) and beta (ERβ). While ERβ has been described as a tumor suppressor, the ERα has been implicated in prostate carcinogenesis and tumor progression^14–16^. Thus, deregulation in estrogenic signaling pathways induced by maternal malnutrition has been associated with impairment of prostate growth in young rats^17^ and higher risk of PCa with aging^18–20^.

Accumulating data have suggested modifications in epigenetic markers as the main mechanistic framework explaining how malnutrition during early life impacts offspring health^21,22^. However, the role of maternal malnutrition on the deregulation of miRNAs-mRNAs networks during prostate development with long-lasting consequences for carcinogenesis remains poorly understood. We hypothesize that maternal malnutrition during pregnancy and lactation changes the offspring's steroidogenic profiles parallel with the deregulation of miRNA–mRNA networks during prostate development, thereby creating fertile soil for slow-growing PCa with aging. To address this hypothesis, we used a rat model of maternal malnutrition to evaluate changes in the steroidogenic pathway and the deregulation of key miRNA–mRNA networks potentially involved in the developmental origin of prostate cancer in old offspring rats. By using this approach, we identified an intricate deregulation of estrogenic signaling pathways and miR-206 networks as important players contributing to the early life origins of PCa in maternally malnourished offspring. Understanding the molecular mechanisms deregulated during prostate development, including epigenetic makers can help us better understand how early life stressors can create a fertile soil for early life origins of prostate cancer throughout the life span.

## Results

### Maternal malnutrition alters biometrical parameters in dams and offspring.

**Figure 1:**
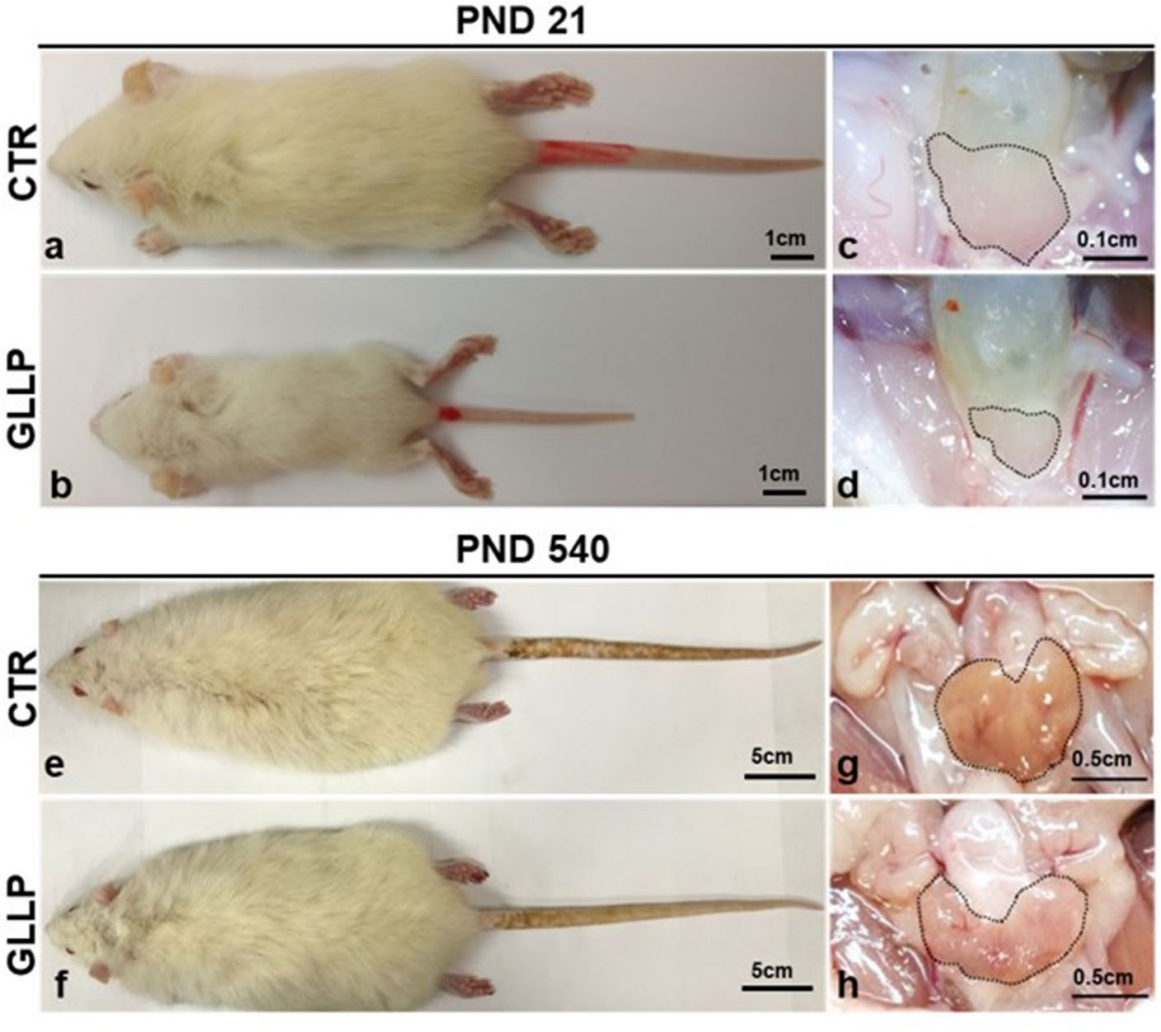
Representative macroscopic images of male offspring (**a**–**b** and **e**–**f**) and prostate (**c**–**d** and **g**–**h**) from Control (CTR) (**c** and **g**) and Gestational and Lactational Low Protein groups (GLLP) (**d** and **h**) at PND 21 (**a**–**d**) and PND 540 (e–h). Note the smaller size of the animals in the GLLP in both ages. Scale bars: (**a**) and (**b**): 1 cm; (**c**) and (**d**): 0.1 cm; (**e**) and (**f**): 5 cm; (**g**) and (**h**): 0.5 cm.

Dams from the GLLP group displayed lower body weight gain during pregnancy and lactation, although the relative food and energy intake did not change compared to the CTR group (Table 1). The offspring from the GLLP group showed a reduced body weight, reduced anogenital distance (AGD) at PND 1 and 21, and lower VP absolute and relative weight at PND 21 compared to the CTR group. At PND 540, it was observed a reduction in offspring body weight in the GLLP group, with no changes in prostate absolute and relative weight and in the AGD (Table 1 and Fig. 1). These data highlighted the key role of maternal diet in alter intrauterine development and growth, with long lasting effects for offspring health.

**Table 1:** Biometric parameters of dams and offspring submitted to low protein diet during gestation and lactation.

Parameters	CTR group	GLLP group
Dams body weight gain during pregnancy (g)	47.25 ± 5.99^a^	37.38 ± 5.02^b^
Dams body weight gain in the lactation (g)	− 0.28 ± 6.70^a^	− 30.44 ± 7.74^b^
Dams energy intake during pregnancy and lactation (KJ/day)	310.30 ± 22.01	326.20 ± 61.38
Number of pups per litter	10.50 ± 1.87	10.29 ± 1.38
Offspring weight at PND1 (g)	7.12 ± 0.55^a^	5.91 ± 0.57^b^
AGD at PND1 (mm)	3.30 ± 0.40^a^	2.89 ± 0.48^b^
Offspring weight at PND21 (g)	37.23 ± 6.57^a^	20.03 ± 3.88^b^
Absolute VP weight at PND21 (mg)	3.24 ± 0.83^a^	1.440 ± 0.39^b^
Relative VP weight at PV 21	0.92 ± 0.19^a^	0.7893 ± 0.16^b^
AGD at PND21 (mm)	10.69 ± 2.55^a^	7.64 ± 1.62^b^
Offspring body weight at PND540 (g)	429.10 ± 51.51^a^	375.0 ± 30.28^b^
Absolute VP weight at PND540 (mg)	627.30 ± 201.20	653.60 ± 226.10
Relative VP weight VP at PND540	1.45 ± 0.37	1.73 ± 0.60
AGD at PND540 (mm)	25.41 ± 3.99	25.01 ± 4.51

The different letters mean statistical difference between experimental groups with *p* < 0.05.

*AGD* anogenital distance; *VP* ventral prostate; *PND* postnatal day; *g* grams; *KJ* kilojoule; *mm* millimeters; *mg* milligram.

### Impairment of prostate growth and imbalance of hormonal levels in young maternally malnouorished offspring

We observed an increase in total cholesterol and plasma concentrations of testosterone and 17β-estradiol in the GLLP group compared to the CTR group at PND 21. Conversely, plasma concentrations of pregnenolone, DHEA, and progesterone were reduced in the GLLP group (Table 2).

**Table 2:** Quantification of serum levels of steroid hormones in animal at PND 21.

Hormone	CTR group (n = 8)	GLLP group (n = 8)
Total Cholesterol (mg/dL)	158.7 ± 25.35^a^	847.8 ± 222^b^
Pregnenolone (pg/mL)	6809 ± 1511^a^	5085 ± 1072^b^
Dehydroepiandrosterone (DHEA) (ng/mL)	16.22 ± 9.43^a^	0.53 ± 0.68^b^
Progesterone (ng/mL)	21.81 ± 11.32^a^	12.28 ± 6.49^b^
17β-hydroxy-4-androstene-3-one (testosterone) (ng/mL)	0.72 ± 0.32^a^	1.84 ± 0.77^b^
17β-estradiol (pg/mL)	16.28 ± 1.45^a^	19.75 ± 3.35^b^

The different letters mean statistical difference between experimental groups with *p* < 0.05.

**Figure 2:**
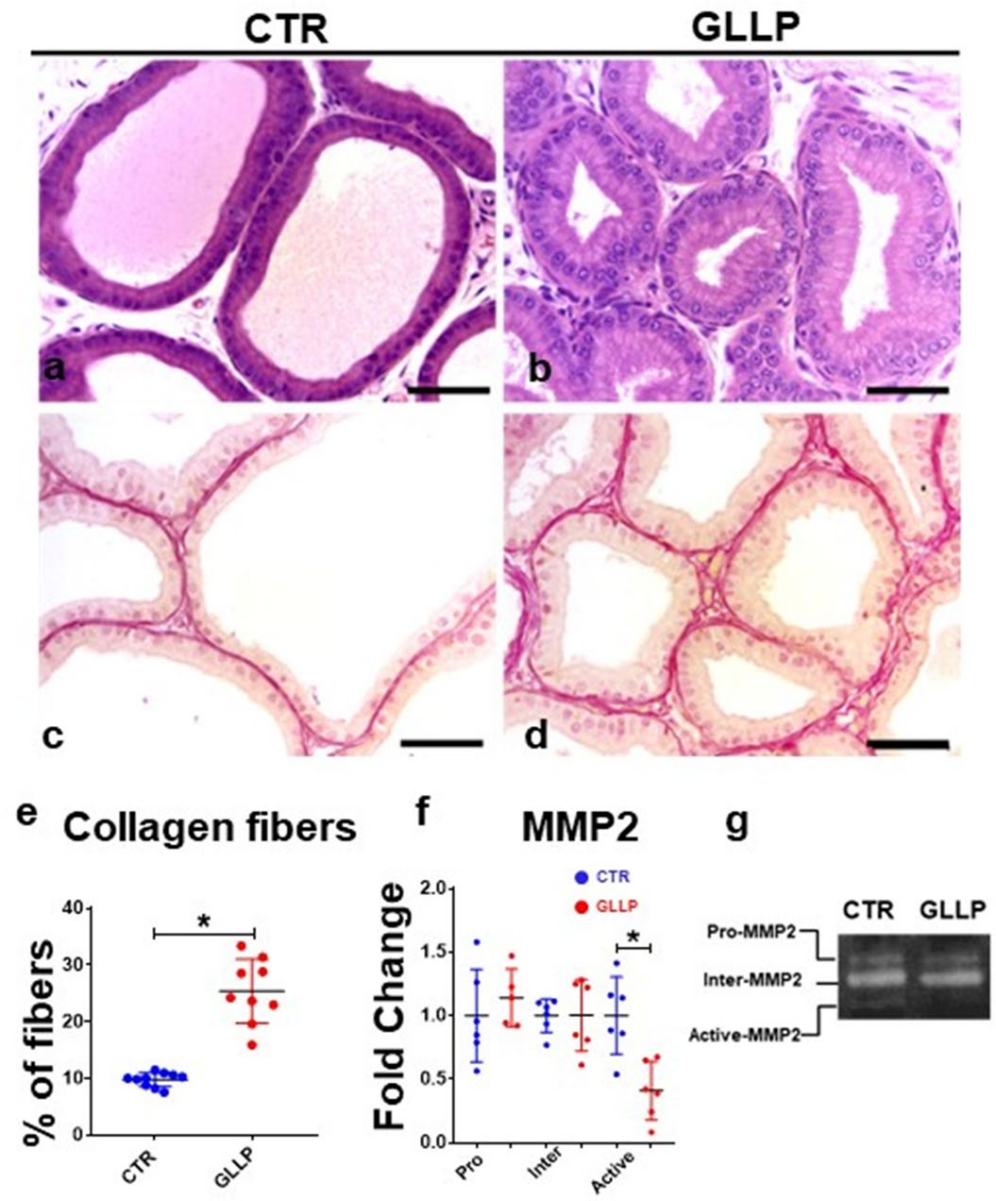
Histological sections of the ventral prostate (VP) lobes from the CTR (**a** and **c**) and the GLLP group (**b** and **d**) stained with hematoxylin–eosin (HE) (**a** and **b**) or picrosirius red (**c** and **d**). Collagen quantification was represented in the bar graph (**e**). Quantification of the VP gelatinolytic activity of pro, intermediate, and active forms of MMP-2 (**f**) in the gelatin zymography gel of electrophoresis (**g**). Data are expressed as mean ± SD. *p < 0.05. Scale bar 50 µm.

The morphological analysis demonstrated an impairment of prostatic growth in the GLLP compared to the CTR group at PND 21 (Fig. 2a and b). We observed a reduction in the lumen of prostatic acini associated with increased collagen deposition in the stromal compartment in the GLLP group compared to the CTR group (Fig. 2c and d). The morphometric data confirmed the increase in collagen content in the stromal compartment (Fig. 2e). Consistently, the gelatinolytic activity of the active form of MMP-2 was reduced in the GLLP group compared to the CTR (Fig. 2f and g, Supplementary Fig. 2).

### Maternal malnutrition deregulates the miRNA–mRNA networks in the young offspring

**Figure 3:**
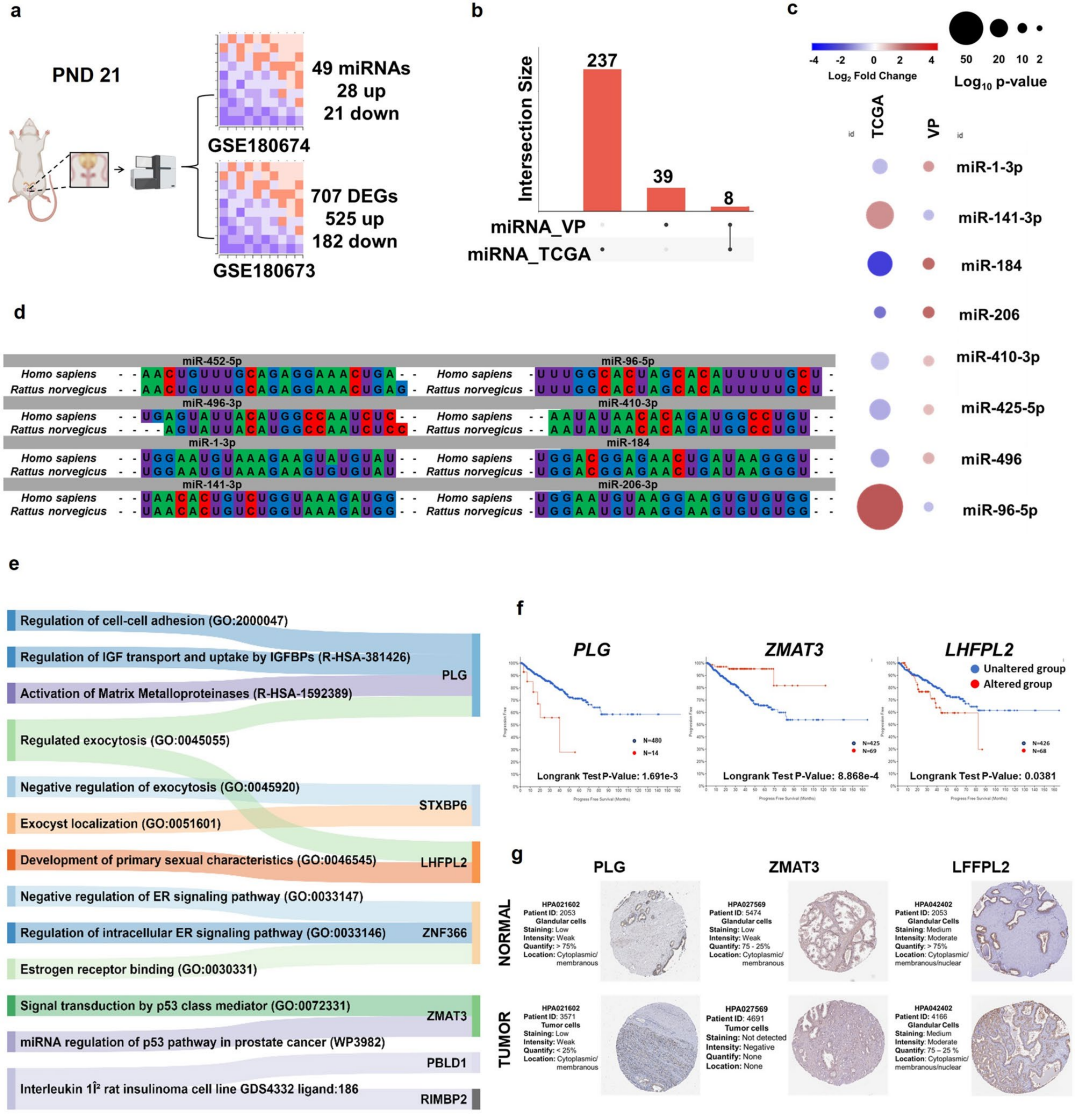
Representative image of bioinformatics analyses. MicroRNome (GSE180674) and transcriptome (GSE180673) datasets from young rat VP were reanalyzed using the DESEq2 package. Differentially expressed targets were considered when *p*-value < 0.05 and Log2 Fold Change ≥|+ 0.66 ≤|− 0.66| (**a**). Up-set plot showing commonly deregulated miRNAs in the VP from the GLLP group and in patients with PCa (**b**). Heatmap showing the miRNAs expression profile shared between the VP from the GLLP group and PCa patients (**c**). Sequence alignment of commonly deregulated miRNAs in the VP from the GLLP group and in PCa samples. The sequences were obtained through the miRBase database (https://www.mirbase.org/) (**d**). Alluvial diagram showing the relationship between the main molecular pathways and enriched ontological terms for the predicted targets of miR-206, these analyzes were performed on the Enrichr platform (https://maayanlab.cloud/Enrichr/), considering *p*-value < 0.05 (**e**). Survival curves of PRAD patients (using TCGA data) show the impact of *PLG*, *ZMAT3*, and *LFPL2* in the progression free PCa patients in altered (red) and unaltered (blue) risk (**f**). Immunohistochemical localization of PLG, ZMAT3, and LFPL2 in normal and PRAD tissues obtained from The Human Protein Atlas database (https://proteinatlas.org/) (**g**).

The integrative analysis identified 49 DE miRNAs (28 up and 21 downregulated) (Supplementary Table 1) and 707 DE mRNA (525 up and 182 downregulated) in the VP from the GLLP group (Supplementary Table 2, Fig. 3a). We evaluated the interaction of 49 DE miRNAs with predicted mRNAs and identified 51,338 potential target mRNAs (Supplementary Table 3). Then, we compared the list of predicted mRNAs with the DE mRNA identified in the VP, resulting in the match of 268 mRNAs potentially regulated by 47 miRNAs (Supplementary Fig. 1 and Supplementary Table 4), considering miRNAs upregulated and mRNA downregulated and vice versa. Collectively, these data demonstrated that maternal exposure to protein restriction deregulated the RNAseq landscape in the VP of young rats, with potential consequences for offspring health.

### The integrative analysis identifies miRNA–mRNA networks and molecular pathways commonly deregulated in the VP of maternally malnourished offspring and patients with PCa

We identified 245 DE miRNAs (118 downregulated and 127 upregulated) in patients diagnosed with PCa using the PRAD-TCGA dataset (Supplementary Table 5). The integrative analysis demonstrated similarities between eight commonly DE miRNAs in the VP from the GLLP group and patients with PCa (miR-1-3p, miR-141-3p, miR-184, miR-206-3p, miR-410-3p, miR-452-5p, miR-496-3p, miR-96-5p) (Fig. 3b and c). Of these, 5 miRNAs (miR-141-3p, miR-96-5p, miR-410-3p, miR-184, and miR-206-3p) shared identical sequences in both human and *Rattus norvegicus* (Supplementary Table 6, Fig. 3d). According to difference in expression profile, sequence conservation and involvement in prostate biology, rno-miR-206-3p and their predicted target mRNAs (*Zfp366*, *Plg*, *Rimbp2*, *Pbld1*, *Lhfpl2*, *Dtna*, *Rmnd5a*, *Zmat3*, *Mob3b*, *Stxbp6*, LOC103692716) were selected for further validation (Supplementary Table 4).

Predicted targets of miR-206 enriched molecular pathways related to the regulation of cell–cell adhesion mediated by cadherin (GO:2000047), regulation of IGF transport and uptake by IGFBPs (R-HSA-381426), activation of matrix metalloproteinases (R-HSA-1592389), regulated exocytosis (GO:0045055), negative regulation of exocytosis (GO:0045920), exocyst localization (GO:0051601) development of primary female sexual characteristics (GO:0046545), negative regulation of intracellular estrogen receptor signaling pathway (GO:0033147), regulation of intracellular estrogen receptor signaling pathway (GO:0033146) estrogen receptor binding (GO:0030331), signal transduction by p53 class mediator (GO:0072331), miRNA regulation of p53 pathway in prostate cancer (WP3982), interleukin IL-1β rat insulinoma cell line (GDS4332 ligand:186). Seven DE mRNAs (*ZNF366*, *PLG*, *ZMAT3*, *RIMBP2*, *PBLD*, *LHFPL2*, and *STXBP6*) were mostly associated with enriched molecular pathways (Fig. 3e).

To further assess the potential role of these seven DE mRNAs in predicting a worse prognosis for PCa (PRAD-TCGA), the cBioPortal tool was used to explore the prognostic index correlating Progression Free Survival analysis and cancer risk assessment. For each gene clustered the PCa patients (n = 494) into two distinct groups (Altered, and Unaltered) (Supplementary Fig. 3). Were associated with poor prognostic the ZMAT3 (*p*-value: 8.868 e−4), LHFPL2 (*p*-value: 0.0381), and PLG (*p*-value: 1.691e−3) (Fig. 3f). Furthermore, data from the HPA database demonstrated changes in the immunolocalization of three targets (PLG, ZMAT3*,* and LHFPL2) in PCa (Fig. 3g). Overall, these results highlight the importance of maternal malnutrition in deregulating early in life key molecular pathways potentially involved in the developmental origins of prostate cancer.

### Transfection of normal prostate cells with mimic miR-206 reduces ESR1 expression, decreases cell viability and migration potential

**Figure 4:**
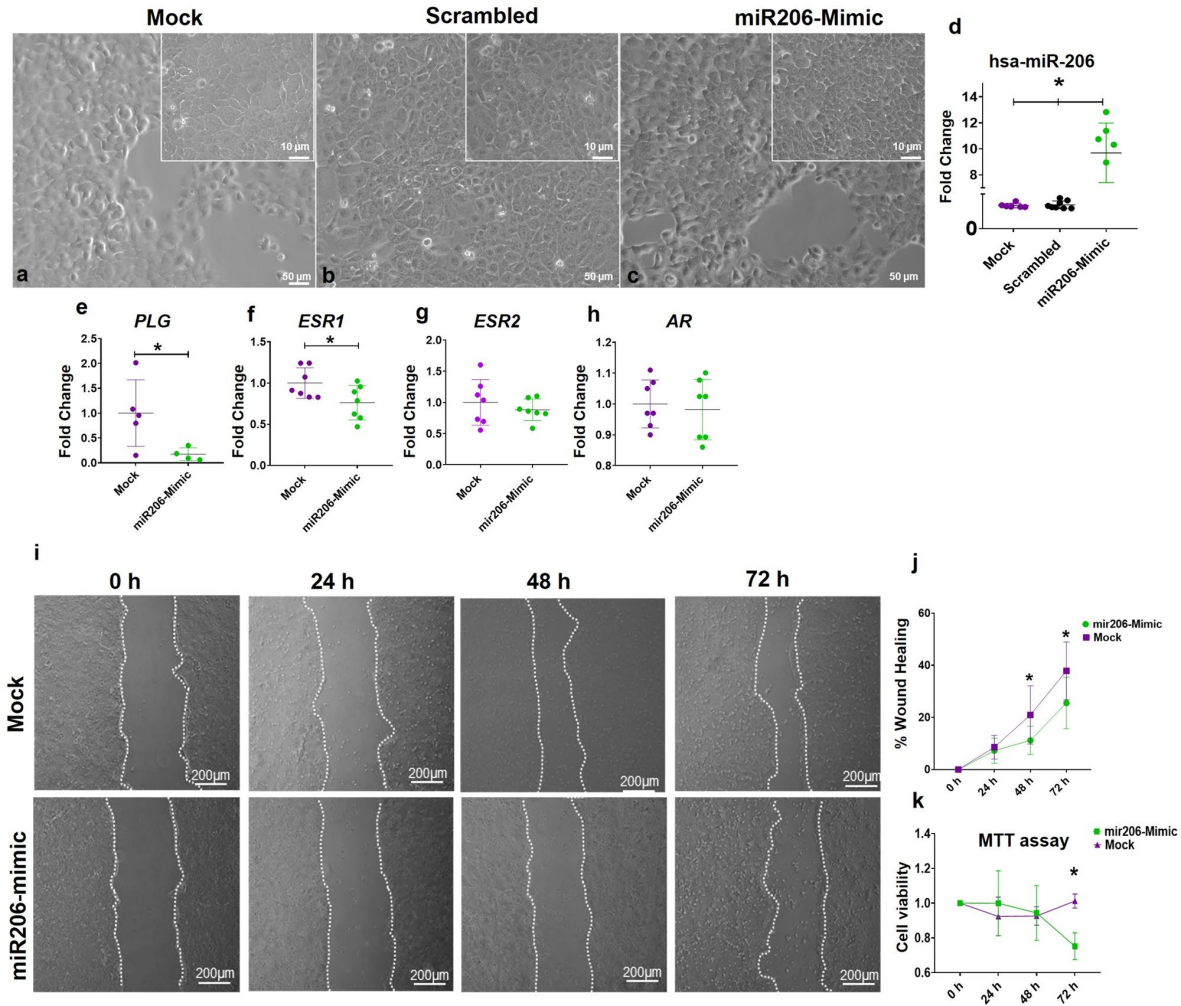
Functional validation of miR-206 in PNT2 benign prostatic cells. Morphologic aspects of the PNT2 cells in the Mock (**a**), Scrambled (**b**), and miR206-Mimic (**c**) groups after 24 h of treatment. Expression profile of miR-206 in PNT2 cells in both experimental groups after 24 h of treatment (**d**). PLG (e), ESR1 (**f**), ESR2 (**g**), and AR (**h**) expression levels in PNT2 cells after 24 h of treatment in both experimental groups. Representative images of wound healing assay after 24, 48, and 72 h (**i**). Cellular wound closure after the transfection of PNT2 cells with Mock and miR206-Mimic groups after 0 h, 24 h, 48 h, and 72 h post-wound (**j**). Cell viability assay (MTT) after 24, 48, and 72 h (**k**). Mock group: cells treated with lipofectamine. Scrambled: cells treated with negative control mimic. miR206-Mimic group: cells treated with miR-206 mimic. Data are expressed as mean ± SD. *p < 0.05. Scale bar: A, B and C 50 µm; detail in A, B and C: 10 µm; j: 200 µm.

After the identification and in silico selection of potential miRNA–mRNA networks altered by maternal malnutrition in the offspring VP, we performed functional assays using benign prostatic PNT2 cells treated with mimic miR-206. Cells transfected with the miR206-Mimic did not show morphological changes compared to cells Mock and Scrambled group (Fig. 4a–c). The miR-206 expression increased in PNT2 cells transiently transfected with miR206-Mimic, while no changes in the miR-206 expression were observed in PNT2 cells treated with Mock or Scrambled group (Fig. 4d). Interestingly, the expression of PLG and ESR1 transcripts decreased in mimic miR-206-treated cells (Fig. 4e and f, respectively), highlighting the potential miR-206-PLG-ESR1 interaction. The expression of ESR2 and AR did not change in this condition (Fig. 4g and h).

The wound-healing assay showed that miR-206 transfected cells delayed the wound healing potential after 48 and 72 h of treatment, compared to the Mock group (Fig. 4i and j). The MTT assays demonstrated a reduction in the PNT2 cell viability after 72 h of exposure to miR-206 mimic compared to the Mock group (Fig. 4k). These data suggest a potential of miR-206 in modulating the prostate cell behavior.

### *Validation of RNAseq and *in vitro* data in the VP of maternally malnourished offspring rats*

**Figure 5:**
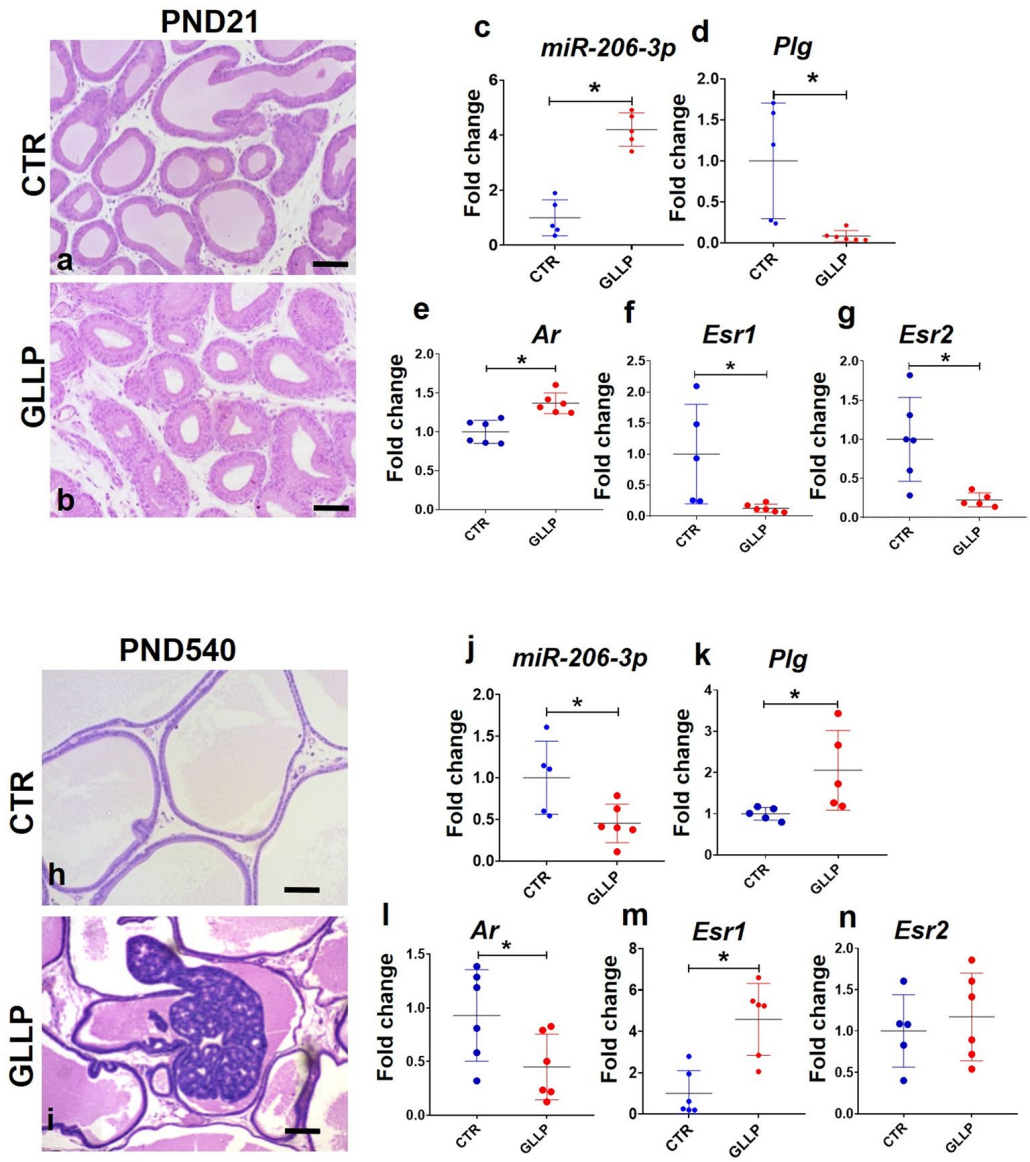
In vivo validation of miR-206/Plg network and steroid hormone receptors (Androgen Receptor: *Ar*; Estrogen Receptor 1: *Esr1*; and Estrogen Receptor 2: *Esr2*) in the VP from the CTR and GLLP groups at PND 21 (**a**–**g**) and 540 (**h**–**n**). Histological sections of the ventral prostate at PND 21 in CTR (**a**) and GLLP (**b**) group showed the impairment of prostate development, and PND 540 in CTR (**h**) and GLLP (**i**) group showing prostate cancer (PCa) in the VP of GLLP group. Representative images of miR-206 (**c** and **j**) and *Plg* (**d** and **k**) gene expression. Hormone receptors gene expression *Ar* (**e** and **l**), *Esr1* (**f** and **m**), and *Esr2* (**g** and **n**) in VP of young and older offspring. Data are expressed as mean ± SD. *p < 0.05. Scale bar: 100 µm.

Consistent with the impairment of the VP growth observed in maternally malnourished offspring (Figs. 5a and b), the RT-qPCR confirmed the upregulation of miR-206 in the VP of young rats from the GLLP group compared to the CTR group at PND21 (Fig. 5c). Interestingly, we also observed that plasminogen expression was reduced in the GLLP group compared to the CTR group (Fig. 5d). Moreover, we also observed an increase in the *Ar* expression and a reduction of *Esr1* and *Esr2* in the VP of young rats from the GLLP group compared to the CTR group (Fig. 5e–g).

To give further insights into the role of epigenetic markers on the developmental origins of prostate cancer (Fig. 5h and i) we demonstrated a reduction in the miR-206 expression in the VP of old rats from the GLLP group compared to the CTR group (Fig. 5j). This was accompanied by an increase in the plasminogen expression levels (Fig. 5k). Moreover, the *Ar* was reduced (Fig. 5l), while *Esr1* was upregulated (Fig. 5m), and the *Esr2* did not change in the VP or old rats from the GLLP group compared to the CTR group (Fig. 5n). Overall, these data demonstrated that maternal protein restriction can be involved in the epigenetic modulation of both androgenic and estrogenic signaling pathways and also modulated the expression profile of important molecular mechanisms involved in the angiogenic process and extracellular matrix remodeling in both young and old offspring VP.

**Figure 6:**
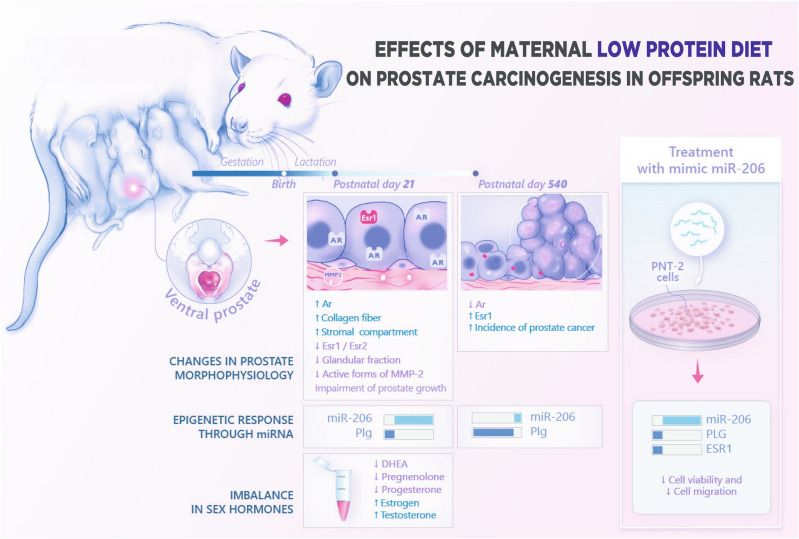
We analyzed and integrated RNAseq data of the ventral prostate to provide new insights into the association of maternal malnutrition with the deregulation of prostate developmental biology with long-lasting effects on prostate health. These analyses revealed that maternal exposure to a low protein diet permanently altered epigenetic markers involved in both prostate growth and carcinogenesis. After target prediction analysis, the deregulation of miRNA 206-ESR1-PLG network was confirmed in both ventral prostate lobes and in human prostate cell culture. Overall, these data, associated with an imbalance in sex steroid hormones observed in male offspring submitted to maternal malnutrition can be implicated in the developmental origins of prostate cancer in maternally malnourished old offspring.

Figure 6 summarizes the workflow depicting the effects of maternal exposure to a low protein diet on the deregulation of the molecular mechanisms involved in the developmental origins of PCa. We further highlighted the key role of the miR-206-network and estrogen signaling pathways (through deregulation of ERα) as a potential prostate cancer driver in older offspring submitted to maternal malnutrition.

## Discussion

Over the past decades, the DOHaD concept has provided causal relationships between early-life exposure to environmental risk factors and the developmental origins of non-communicable chronic diseases, including PCa^8,18,19^. Although the molecular mechanisms involved in this process remain elusive, the imbalance of sex hormones in both dams and offspring submitted to maternal malnutrition has been pointed to as a key factor related to prostate carcinogenesis in the progeny^5,8,9,18,19^.

To further elucidate how maternal malnutrition interferes with the balance of sex hormones in male offspring, we evaluated the serum cholesterol profile and their intermediate metabolites involved in the steroidogenesis pathways. An increase in total cholesterol was observed in GLLP animals at PND 21. Sohi et al. (2011) have demonstrated an increase in cholesterol levels in male rat offspring submitted to MPR (8% protein) at the same age and associated this result with permanent epigenetic silencing of the hepatic *Cyp7a1* promoter, which metabolizes cholesterol to bile acids^23^. The offspring from the GLLP group also showed an imbalance in plasma concentrations of steroid hormones; while testosterone and estradiol were elevated, pregnenolone, progesterone, and DHEA were reduced, suggesting that these intermediate metabolites could be consumed to sustain the higher levels of testosterone and estradiol.

Although largely driven by androgens, the developing prostate is sensitive to estrogens. Epidemiological and experimental data have demonstrated that abnormal exposure to an excessive estrogenization early in life may disrupt molecular mechanisms governing prostate developmental biology, with long-lasting consequences for prostate diseases with aging^10,18,19^. As such, African-American men are at higher risk of prostatic carcinoma than their Caucasian counterparts, and it has been postulated that this higher risk is related, in part, to the higher estrogens levels observed during pregnancy in the former population^9,10^. These conditions, termed “developmental estrogenization” or “estrogen imprinting”, states that inappropriate exposure to estrogen (due to ethnicity, exposure to the endocrine disruptor, environmental chemicals, maternal malnutrition, among other individual and environmental factors) is memorized (“imprinted”) by cells and tissues^11,12,14,24^. Understanding the relationship between exposure to an estrogenic environment with long-lasting consequences for prostate diseases is essential for the DOHaD concept. It has been demonstrated in rodent models that exposure to high levels of estrogens early in life blocks epithelial cell differentiation and increases the number of basal epithelial cells^17^. Using proteomic approaches, Santos et al. 2020 identified estrogenic signaling pathways, endoplasmic reticulum functions, oxidative stress, and deregulation of insulin/IGF signaling pathways as the main deregulated mechanisms involved in the VP response to maternal malnutrition in both young and older male offspring^22^. We further demonstrated that the impairment of the glandular compartment in young rats was associated with an increase in collagen fiber deposition in the stroma. The increase in collagen content can be explained, at least in part, by the reduction of the gelatinolytic activity of the active form of the MMP-2, a class of enzyme responsible for extracellular matrix remodeling in the prostate gland^25^.

To give further translational insights into the relationship between deregulated miRNAs networks early in life and the developmental origins of PCa, we identified 5 miRNAs with similar nucleotide sequences commonly deregulated in the VP of maternally malnourished offspring and patients with PCa. These miRNAs have been associated with tumorigenesis and aggressiveness of several types of cancers, including miR-141-3p which appeared deregulated in breast and prostate cancers^22,26,27^. The miR-96-5p was associated with aggressiveness and metastatic behavior of ovarian^28^, breast^29^, and prostate cancers^30^. The miR-410-3p has a central role in cancer-associated cachexia^31^, and the reduction of miR-410-3p expression was correlated to the poor prognosis in colorectal cancer^32^. The miR-184 was described as the tumor suppressor, controlling cell cycle, proliferation, and apoptosis process, being reported in carcinogenesis processes in non-small cell lung cancer (NSCLC)^33^, and prostate cancer^34^. Regarding miR-206, although this miRNA has been described as a tumor suppressor miRNA, that modulates cell proliferation, migration, and aggressiveness in various types of cancer, including prostate cancer^35,36^, recently it has been demonstrated that miR-206 can act as an important “DOHaD miR”^37^. In this study, Yamazaki et al. (2020) showed that maternal exposure to fructose resulted in deregulation of the miR-206-Lxra network in the liver offspring rat and associated this result with a reduction in serum HDL-C level and metabolic syndrome in these animals^37^. Considering the potential role of miR-206 related to the early origins of PCa, we select this miRNA for further experimental validation.

RT-qPCR confirmed the upregulation of this miRNA in the VP of young offspring. Accordingly, experimental upregulation of miR-206 led to retardation of mammary gland development through modulation of *Wnt* and transcription factors *Tbx3* and *Lef1*^38^. The upregulation of miR-206 in the GLLP group can be associated with stromal collagen accumulation in the impaired VP of young rats since the miR-206/*Plg* network, which modulates the extracellular matrix remodeling and angiogenesis processes^39,40^, was downregulated in these animals.

It is noteworthy that the prostate is particularly more sensitive to estrogens during critical windows of development^11^. Estrogen actions on the prostate are mainly mediated through major canonical estrogen receptors (ER), including ERα and ERβ. Studies involving knockout mice and developmental exposure to estrogenic compounds indicate that paracrine signaling involving stromal ERα is the dominant form of estrogen-mediated imprinting in the developing, mammary^41^ and prostate gland^14,15^. Consistently, the deletion of the ERα gene in the stromal fibroblast leads to a reduction in prostate branching morphogenesis^42^. Additionally, Lee et al. (2013) demonstrated that miR-206 epigenetically downregulates *ESR1* expression during normal mammary gland development^38^. Although there is no data regarding the *Esr1* modulation by miR-206 in the developing prostate, our data demonstrated the potential relationship of miR-206 in modulating ER signaling since *miR-206*-ERs networks have opposite expression profiles in the VP of young offspring in the GLLP group.

In addition to the potential role of miR-206 as an important modulator of gene expression during the developmental process, a body of evidence highlighted its key role as a tumor suppressor miRNA in PCa^35,43^. However, there is no data regarding the epigenetic modulation of ERs by miR-206 in PCa. Studies with knockout mice for estrogen receptors indicate that *Esr1* and *Esr2* can have opposite actions on prostate carcinogenesis since *Esr1* is recognized to promote cell proliferation, while *Esr2* has been described as anti-proliferative and proapoptotic. Altogether, these results suggest that proliferative response to estrogen results from a balance between ERα and ERβ signaling^16,44,45^. Our functional validation experiments demonstrated downregulation of *ESR1* expression in PNT2 cells transfected with miR-206. These results are consistent with deregulation of the miR-206*-ESR1* network observed in the VP of young and older offspring rats from the GLLP group. Moreover, it has been demonstrated that in vitro inhibition of miR-206 in PC-3 cells increases cell invasion through upregulated ANXA2 and E-cadherin, and downregulation of N-cadherin and vimentin^43^. Wang et al. (2018) also described the tumor suppressor effect of miR-206 in PCa by negatively regulating cell proliferation and migration by targeting *CXCL11*^34^. These authors also demonstrated experimentally that up-regulation of miR-206 inhibits proliferation, migration, invasion, and induced G1/G0 arrest of PCa cells. The expression profile of miRNA extracted from PRAD-TCGA demonstrated the downregulation of miR-206 in patients with PCa. Interestingly, we observed a reduction in the expression profile of miR-206 and an increase in *Plg* gene expression in the VP of older offspring rats from the GLLP groups, which developed prostate carcinoma *in situ*^18,19^. Functional validation confirmed the modulation of plasminogen expression by miR-206, as well as their participation in modulating cell migration and invasion in PNT2 cells. Furthermore, we provide new evidence on the participation of miR-206-ESR1 network mediating prostate carcinogenesis in maternally malnourished offspring rats.

Although there are some similarities between molecular pathways controlling prostate developmental biology and carcinogenesis^46^ it is important to emphasize that the deregulation of miR-206 networks in young and old offspring rats can impact differently prostate growth and carcinogenesis. In young offspring rats, the upregulation of the miR-206 and downregulation of their target mRNAs and molecular pathways (such as decreased activity of the estrogen receptor, p53, and IGF/Insulin pathways) are associated with delay in prostate growth in maternally malnourished offspring rats. On the other hand, the opposite modulation of the miR-206 in old offspring is thought to act in favor of prostate carcinogenesis, as IGF/insulin and estrogen signaling pathways, mainly mediated by estrogen receptor alpha (ERS1) are classically described as procarcinogenic pathways^47^. Thus, we believe that the deregulation of miR-206 networks throughout the lifespan can contribute to the developmental origins of prostate carcinogenesis, as demonstrated previously by our research group^18^.

Overall, our data provide new insights into the participation of an estrogenized environment associated with deregulation of miR-206-PLG-ESR1 in the developmental origin of prostate diseases in maternally malnourished offspring rats. Understanding these mechanisms can encourage the adoption of a governmental policy for the early life prevention of non-communicable chronic diseases, as proposed by the DOHaD concept.

## Materials and methods

### Animals and diets

Naive adult (90 days age) females (n = 30) and males (n = 10) Sprague Dawley rats were used. The animals were kept under a controlled temperature (22–25 °C), relative humidity (55%), and a photoperiod (12 h/12 h), with free access to water and food. Breeding proceeded overnight in a harem configuration (1 male to 3 females). After determination of pregnancy through detection of spermatozoa in the vaginal smear (considered gestational Day 1—GD1), pregnant rats were distributed in Control group (CTR, n = 15): Pregnant rats fed with a normal protein diet (17% protein) during the gestation and lactation; and Gestational and Lactational Low Protein group (GLLP, n = 15): Pregnant rats fed with a low protein diet (6% protein) during the same periods. The diets followed the AIN-93 standards described by Reeves et al. (1993) and were provided by PragSoluções (PragSoluções, SP, Brazil)^48^ (Supplementary Table 7). The diets were previously used in other studies^17–19,22^. Litters were reduced to eight pups (four males and four females) at a postnatal day (PND) 1 to maximize lactation performance^49^. Maternal body weight and food intake were recorded every 3rd day until GD 21. Dam and offspring biometric parameters were measured until the end of the experiment. One male offspring (21 days old) from each litter was euthanized by an overdose of ketamine and xylazine, followed by decapitation. Blood and ventral prostate (VP) were collected and processed as described below. The biometric parameters of old rats were generated in a previous study of our research group ^21^. All experiments were performed in accordance with the Ethics Committee for Animal Experimentation at the Institute of Biosciences/UNESP (Protocol #1178). Throughout the experiment, housing and use of animals were performed accordingly with the appropriate guidelines and regulations. Efforts were made to minimize suffering and to reduce the animal numbers used in the experiments. The acquisition and description of data followed the recommendations set out in the ARRIVE guidelines.

### Serum blood hormonal analysis

Blood samples from male offspring (n = 8/group) were centrifuged (2400 g for 20 min), and were used to quantify total cholesterol (Labtest, R76-2/100, Brazil, sensitivity: 0.06 mg/dL), pregnenolone (LifeSpanBioSciences, LS-F39295, USA, sensitivity: 9.375 pg/mL); dehydroepiandrosterone (DHEA) (Monobind, CA 7425-300, USA, sensitivity: 0.10 ng/mL), estrogen (17βestradiol, Monobind, CA 4925-300, USA, sensitivity: 8.2 pg/mL) and testosterone (17β-hydroxy-4-androstene-3-one, Monobind, CA 3725-300A, USA, sensitivity: 0.038 ng/mL). The hormonal quantifications were made in 96-well plates using the ELISA plate reader (Epoch, Biotek Instruments, VT, USA) following the manufacturer's protocol.

### Morphology analysis and gelatin-zymography analyses

Samples of VP lobes from CTR and GLLP groups (n = 12/group) at PND 21 were processed for histological analysis as described by Santos et al. (2019)^18^. The slides (5 µm) were stained with Hematoxylin/Eosin or with picrosirius red. The slides were analyzed using the image analyzer Leica Q-win software (Version 3 for Windows) coupled to a Leica DMLB 80 microscope (Leica, GER). The collagen fiber volume was determined by a red color automatic detection in 10 different microscopic fields (200X) from 6 different VP lobe sections. The collagen volume was shown as a percentage of red-stained areas per total prostatic area.

The gelatin zymography was performed as described in Justulin et al. (2010)^25^. Briefly, frozen VP lobes (n = 6/group) were homogenized in RIPA buffer (50 mM of Tris buffer pH 7.5 plus 0.25% Triton-X 100), centrifuged, and the total proteins were quantified by the Bradford method^50^. Aliquots (28 μg protein) were subjected to electrophoresis in 0.1% gelatin-containing polyacrylamide gels (8% acrylamide) in a Bio-Rad MiniProtean II system (Bio-Rad Laboratories Inc., Richmond, CA, USA). Next, the gels were shaken with 2.5% Triton-X100 and incubated overnight in 50 mM Tris–HCl (pH 8.4) containing 5 mM CaCl_2_ and 1 μM ZnCl_2_ at 37 °C. Then, the gels were stained with Coomassie Blue, and areas of proteolysis were measured using ImageJ software (National Institutes of Health, USA). The results were expressed in fold change as the mean ± SD.

Briefly, the histopathological analysis was performed only in old offspring rats (n = 12 per group) by the pathologist WRS following the criteria described by Bernoulli et al. (2008)^51^ and Bosland et al. (1998)^52^. Briefly, four histological sections (per animal) were collected in the range of 200 μm and stained with Hematoxylin and Eosin. The prostate carcinoma in situ was classified as follows: localized carcinoma, loss of cell polarity, presence of nuclear polymorphism, microacini occupying the acinar lumen, and microangiogenesis circumscribing into the prostatic acini.

### Identification of deregulated miRNAs-mRNAs networks in offspring VP

To identify the potential deregulation in miRNAs-mRNAs networks in the VP of young rats, we reanalyzed data of next-generation sequencing (NGS) from the CTR (n = 4) and GLLP (n = 3) groups. Briefly, total RNA was extracted using Trizol (Ambion, USA) following the manufacturer's instructions. Total RNA was quantified using the NanoDrop (Thermo Scientific, USA) and the RNA Integrity Number (RIN) was determined using the 2100 Bioanalyzer system (Agilent, USA). Only RNA samples with RIN > 8 were used. Libraries were treated with Ribo-zero for rRNA depletion, and RNAs were separated by size using TruSeq Stranded mRNA Sample Preparation and TruSeq Small RNA Library Preparation kits (Illumina, USA). Sequencing was performed using the HiSeq Sequencing System (HiSeq2500, Illumina). Raw data were downloaded, the quality of reads was measured by FASTQc, and adapter sequences were removed using Trimmomatic^53,54^. The identification of miRNAs and mRNAs was performed using the Spliced Transcripts Alignment to a Reference (STAR) tool^55^. The genome of Rattus novergicus (Rnor_6.0) and its annotation files were used as references. These RNAseq data were available for download at the Gene Expression Omnibus (GEO) database under accession numbers GSE180674 (miRNA) and GSE180673 (mRNA). The differentially expressed (DE) miRNAs and mRNAs were identified using the DESeq2 package (https://bioconductor.org/packages/DESeq2/)^56^. The cut-off significance for miRNA and mRNA was *p*-value < 0.05 and Log2 Fold Change ≥|+ 0.66 ≤|− 0.66|.

After identifying deregulated miRNAs in the rat VP, the predicted target mRNAs were identified using miRWalk 3.0 platform (http://mirwalk.umm.uni-heidelberg.de/) for *Rattus norvegicus*^57^. The cut-off criterion for significance was *p*-value < 0.05. To further identify the deregulated miRNAs-mRNAs network potentially involved with prostatic disorders, the list of predicted mRNAs was integrated with the differentially expressed (DE) mRNAs identified in our transcriptome dataset, considering miRNAs upregulated and mRNA downregulated and vice versa.

### Identification of miRNAs commonly deregulated in the offspring VP and human PCa

To identify DE miRNAs in patients with PCa, we explored the Prostate Adenocarcinoma dataset (PRAD-TCGA, https://portal.gdc.cancer.gov/projects/TCGA-PRAD), with data from 550 samples (52 normal samples and 498 tumor samples). The DE miRNAs were identified using the DESeq package^56^. The cut-off significance for both miRNA and mRNA were *p*-value < 0.05 and Log_2_ Fold Change ≥|+ 0.66 ≤|− 0.66|. Then, we compared the common DE miRNAs in the VP of maternally malnourished offspring with those from patients with PCa.

### Criteria for selection of miRNA commonly deregulated in the offspring VP and human PCa

To select a miRNA commonly deregulated between VP and human PCa for further analysis, we took into account the higher fold change observed in our RNAseq dataset, the miRNA conservation sequences between *Rattus norvegicus* and human (checked through the miRBase database, http://www.mirbase.org/)^58^, and their role in prostate biology^35,43^.

### *Identification of selected miRNA–mRNAs networks on human PCa: *in silico* approach*

To further explore the role of selected miRNA–mRNAs networks on PCa, the mRNAs predicted to be regulated by the selected miRNA were subjected to enrichment analysis of molecular pathways, biological processes, and cellular components using the Enrichr tool (https://maayanlab.cloud/Enrichr/)^59^. Subsequently, these mRNAs were applied in overall survival analysis and risk assessment in PCa patients using the cBioPortal platform (https://www.cbioportal.org/)^60^. The Human Protein Atlas (HPA) (https://www.proteinatlas.org/) database was used to demonstrate the immunolocalization of deregulated mRNAs in human prostatic samples^61^.

### Functional validation of selected miRNA in transfected prostatic cell line

The benign prostate cell line (PNT2, Cell Bank of Rio de Janeiro, Brazil) was cultured using RPMI 1640 medium supplemented with 2 mM L-glutamine (GIBCO, 31800-022, USA), 10% FBS (Nova Biotecnologia, 10-bio500i, Brazil), 50 μg/mL penicillin–streptomycin, and 0.5 μg/mL amphotericin B (GIBCO, 15240-062, USA). The cells were grown at 37 °C with 5% CO_2_ in a humid atmosphere and used up to passage 20. The culture medium was changed twice a week, and throughout the experimental period, the cells were monitored with an inverted microscope (Zeiss Axiovert, USA). For passage, the cells were released with 0.25% trypsin (GIBCO, 25200-072, USA) for 5 min at 37 °C, resuspended in a fresh medium, and plated again. Based on the significance of enriched terms (*p* < 0.05) and its relevance for prostate biology, mRNA was selected for validation through RT-qPCR.

### miRNA transfection and cell viability assay

For in vitro functional validations, PNT2 cells were divided into 3 experimental groups: Mock (PNT2 cells treated with lipofectamine), miR206-Mimic (PNT2 cells transfected with mimic miR-206) and Scrambled (PNT2 cells transfected with non-specific mimic control). Before transfection, the PNT2 cells (8 × 10^4^) were plated in 12-well plates in 800 μl of complete RPMI medium per well. Once cells became 80% confluent, transfections in all experimental groups were performed using a complex with the Opti-MEM reduced serum medium (Thermo Fisher Scientific, 31985-062, USA) and RNAiMAX lipofectamine (Thermo Fisher Scientific, L13778-150, USA) with or without 10 nM of specific mimic miRNA (mirVana miRNA Mimic, code: 4464066 MC10409, Thermo Fisher, USA) or Negative control (mirVana miRNA Mimic, Negative Control #1, code: 4464058 AS02D00R, Thermo Fisher, USA) for 16 h at 37 °C and 5% CO_2_. After 24, 48, and 72 h, cell viability was determined by the MTT reduction method according to the manufacturer's instructions (Invitrogen, M6494, USA)^62^. As absorbance is proportional to cell viability, the percentage of cell viability relative to control cells was quantified in a spectrophotometer (ASYS HITECH GmbH, AUT) using a 96 well plate at 550 nm absorbance.

### Wound healing assay

PNT2 cells were cultured in a 6 well plate using RPMI 1640 medium at 37 °C and 5% CO_2_ until reached 70–80% confluence. The cells were transfected with RNAiMAX lipofectamine with or without 10 nM of specific miRNA mimic (miR206-Mimic and Mock groups, respectively) for 16 h at 37 °C and 5% CO_2_. After, a 200 μL plastic tip was used to perform a single-line scratch at the highest diameter in all replicates (T0h). Cell debris was removed using two PBS washes, and 1.2 mL RPMI was added to each well. The analysis of the wound open area was performed at 0, 24, 48, and 72 h and the results were expressed as a percentage of wound healing, following the equation: % wound healing = [100 − (wound area at Tnh/wound area at T0h)] × 100, where T0h is the time point immediately after the scratch.

### Expression profile of selected miRNA–mRNA network after transfection

Total RNA of PNT2 cells from Mock, mimic206-Mimic, and Scrambled groups was extracted using TRIzol (Thermo Fisher Scientific, 15596026, USA) as recommended by the manufacturer. For miRNA reactions, the High-Capacity RNA-to-cDNA Kit (Life Technologies, 4387406, USA) was used for cDNA synthesis from 100 ng of total RNA according to the manufacturer's recommendations. Stem-loop RT primers were synthesized according to Chen et al. (2005)^63^. The cDNA was amplified with universal and forward primers, and the quantifications were normalized using the reference miRNA (U6).

To determine the expression profile of selected mRNAs, aliquots of 2 μg of total RNA were reverse transcribed using the High-Capacity Kit RNA-to-cDNA (Life Technologies, 4387406, USA) in a 10 μL reaction according to the manufacturer's instructions. Aliquots of cDNA from each sample were added to a mix of reagents containing primers “sense” and “anti-sense,” and the volume was completed to 10 µL with ultrapure water. Primers were designed specifically for target genes after in silico filtering. The values obtained for all samples were normalized by the ratio between the target genes and the reference genes (*ACTIN* and *GAPDH*). For these experiments, the cells from the Mock group were used as the control group.

All reactions were used using the SYBR Green PCR Master Mix system (Thermo Fisher, 4309155, USA). The expression level of the selected miRNA and mRNAs was determined using the QuantStudio 12K Flex real-time PCR system (Thermo Fisher Scientific, USA) on 96 plate wells. The values were calculated using the expression ratio of the miR206-Mimic/Mock groups. The relative quantification of each gene was performed using the 2^−∆∆CT^ method normalized using^64^. The sequences of the primers were designed on the Primer Express 3.0 software (Supplementary Table 8).

### Validation of the selected targets in the young and old offspring VP

The total RNA samples from the young (PND21) and old (PND540) VP offspring rats submitted to maternal malnutrition (n = 6/group in both ages) were used in these analyses. The RNA samples of old rats were generated in a previous study from our research group^18^. The validation of the miRNA, and mRNA expressions followed the protocol described in item 2.8.3. Primer sequences for *Rattus norvegicus* were described in Supplementary Table 8.

### Statistical analysis

Statistical analyses were performed using the GraphPad Prism software (version 5.00, Graph Pad, Inc., San Diego, CA). The results were submitted to normalization analysis (Shapiro–Wilk test). Parametric data were compared using the Student’s t-test. The remaining (dams body weight gain at lactation period, offspring weight at PND21, DHEA concentration, and *Plg* expression in VP samples) were submitted to the Mann–Whitney test. The miR-206 expression data in transfected cells were subjected to analysis of variance (ANOVA), followed by the ‘‘Tukey–Kramer” test. Data were expressed as mean ± SD. The differences were considered statistically significant when *p* < 0.05. The statistical tests used are described in the figure legends.

### Data representation and analyses

Bar graphs were generated using the GraphPad Prism tool (GraphPad Software). The heatmap with the results of RT-qPCR was created using the Morpheus web tool (https://software.broadinstitute.org/morpheus)^65^. miRNA–mRNAs networks were demonstrated by circus plot graphics generated in the R environment using the “Circlize” package^66^. The UpSet plot generated to represent deregulated miRNAs in the VP of animals submitted to maternal malnutrition and PCa patients was made on the Intervene platform (https://intervene.shinyapps.io/intervene/)^67^. The relationships between the molecular pathways and the predicted targets of miRNA, demonstrated in an alluvium diagram, were performed on the Sankeymatic platform (https://sankeymatic.com/).

### Ethics approval

This study was performed in line with the principles ethics. Approval was granted by the Biosciences Institute/UNESP Ethics Committee for Animal Experimentation (Protocol #1178).

### Supplementary Information

Supplementary Information.

Supplementary Tables.

## Data Availability

The data that support the findings of this study are openly available in The Gene Expression Omnibus (GEO) (https://www.ncbi.nlm.nih.gov/gds) on accession number(s) GSE180673 and GSE180674. Other data that support the findings of this study are available in The Cancer Genome Atlas (TCGA) at (https://portal.gdc.cancer.gov/projects/TCGA-PRAD). These data were derived from the following resources available in the public domain: https://portal.gdc.cancer.gov/projects/TCGA-PRAD; http://bioinformatica.mty.itesm.mx:8080/Biomatec/SurvivaX.jsp.
