# Supplementary material for: Early-life origin of prostate cancer through deregulation of miR-206 networks in maternally malnourished offspring rats
Source: Sci Rep. 2023 Oct 31;13:18685. doi: 10.1038/s41598-023-46068-1 (PMC10618455; doi:10.1038/s41598-023-46068-1)

**Early-life origin of prostate cancer through deregulation of miR-206 networks in maternally malnourished offspring rats**

Luiz MF Portela (0000-0002-5898-3615)¹; Flavia B Constantino (0000-0002-8831-325X)¹; Ana CL Camargo (0000-0003-4993-2573)¹; Sérgio AA Santos (0000-0002-1375-1634)¹^,4^; Ketlin T Colombelli (0000-0002-2711-6359)¹; Matheus N Fioretto (0000-0003-0138-7959)¹; Luísa A Barata (0000-0003-0018-720X)¹; Erick JR Silva (0000-0002-9330-8658)²; Wellerson R Scarano (0000-0002-6682-2934)¹; Sergio L Felisbino (0000-0002-6870-5192)¹; Carlos S Moreno (0000-0002-5582-0028)^3^; Luis A Justulin (0000-0002-7358-3302)¹

¹ Department of Structural and Functional Biology, Institute of Biosciences, Unesp Botucatu, Brazil

² Department of Biophysics and Pharmacology, Institute of Biosciences, Unesp Botucatu, Brazil

^3^ Department of Pathology and Laboratory Medicine, Emory University School of Medicine, Atlanta, Georgia, USA; Department of Biomedical Informatics, Emory University School of Medicine, Atlanta, Georgia, USA

^4^ Cancer Signaling and Epigenetics Program, Fox Chase Cancer Center, Philadelphia, PA 19111, USA

**Corresponding author:** Luis A Justulin

Sao Paulo State University (UNESP), Institute of Biosciences of Botucatu, SP, Brazil.

**Zip Code:** 18618-689

**Phone number:** +551438800481

**email**: l.justulin@unesp.br

**Supplementary Material**

**Supplementary Figure 1.** Identification of deregulated miRNAs-mRNAs netorwks in the VP of the GLLP group. Circos plot showed miRNA-mRNA interactions; 268 DEGs are commonly targeted by 47 miRNAs. Ribbons start from a miRNA (lower) and end in an mRNA (upmost). The color of the ribbons indicates the originating miRNAs.

**Supplementary Figure 2.** Representative image of zymography gel electrophoresis in the ventral prostate (VP) from Control (CTR) and Gestational and Lactational Low Protein groups (GLLP).

**Supplementary Figure 3.** Survival curves of PRAD patients (using TCGA data) show the impact of PBLD, RIMBP2, ZNF366, and STXBP6 in the progression free PCa patients in altered (red) and unaltered (blue) risk

**Supplementary Table 1.** Composition of the feed or Normoprotein and Low protein diet (AIN-93).

**Supplementary Table 2.** Sequence design of the primers that were used in the RTq-PCR reactions.

**Supplementary Table 3.** Biometric parameters of dams and offspring submitted to low protein diet during gestation and lactation.

**Supplementary Table 4.** Quantification of serum levels of steroid hormones in animal at PND 21.

**Supplementary Table 5.** miRNA data differentially expressed in VP of animals submitted the maternal malnutrition at DPN21 (GSE180674)

**Supplementary Table 6.** mRNA data differentially expressed in VP of animals submitted the maternal malnutrition at DPN21 (GSE180673)

**Supplementary Table 7.** Predicted targets of miRNAs in the PV of animals submitted to maternal malnutrition

**Supplementary Table 8.** Integration of the network of miRNAs and predicted mRNAs in the PV of animals submitted to maternal malnutriton

**Supplementary Table 9.** miRNA expression data in patients with prostate cancer (PCa) (PRAD-TCGA data)

**Supplementary Table 10.** Sequences of miRNAs in humans and rats

**Link for supplementary data**

<https://drive.google.com/drive/folders/1uSZd5X1I5vWbgi1gITMn1Kom3cjq4z6a?usp=sharing>

**Supplementary Figure 1.**


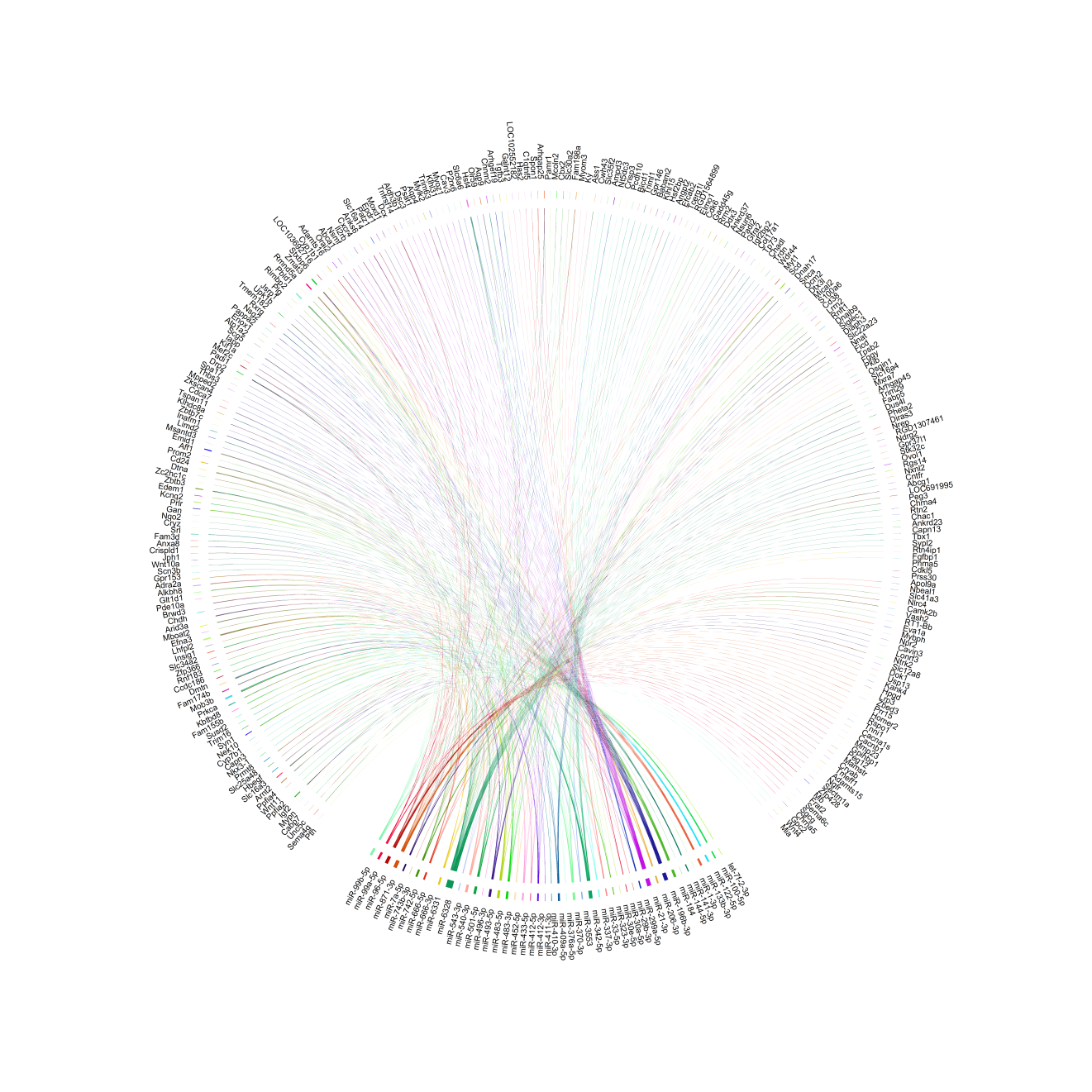


**Supplementary Figure 2.**

**
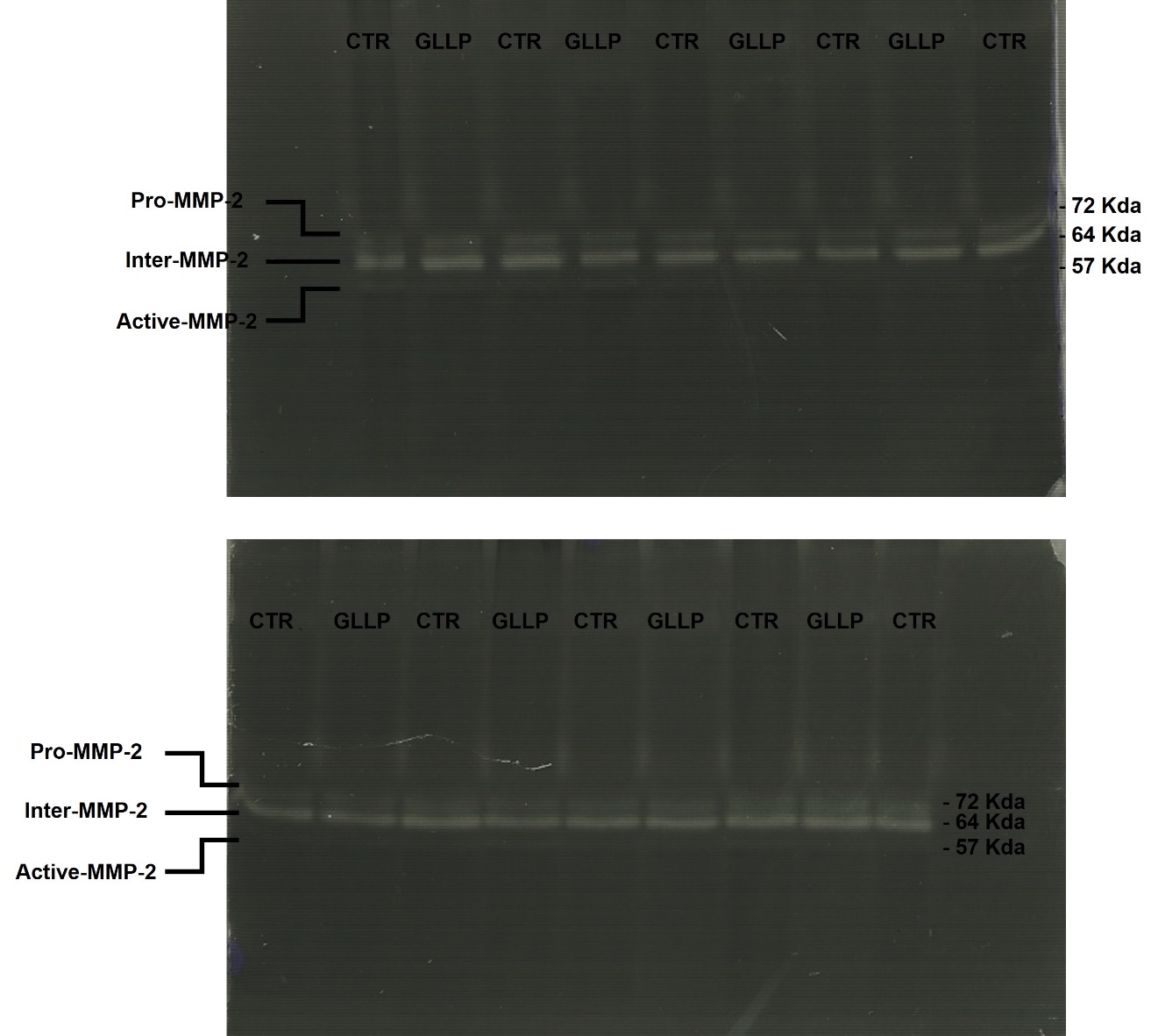
**

**Supplementary Figure 3.**


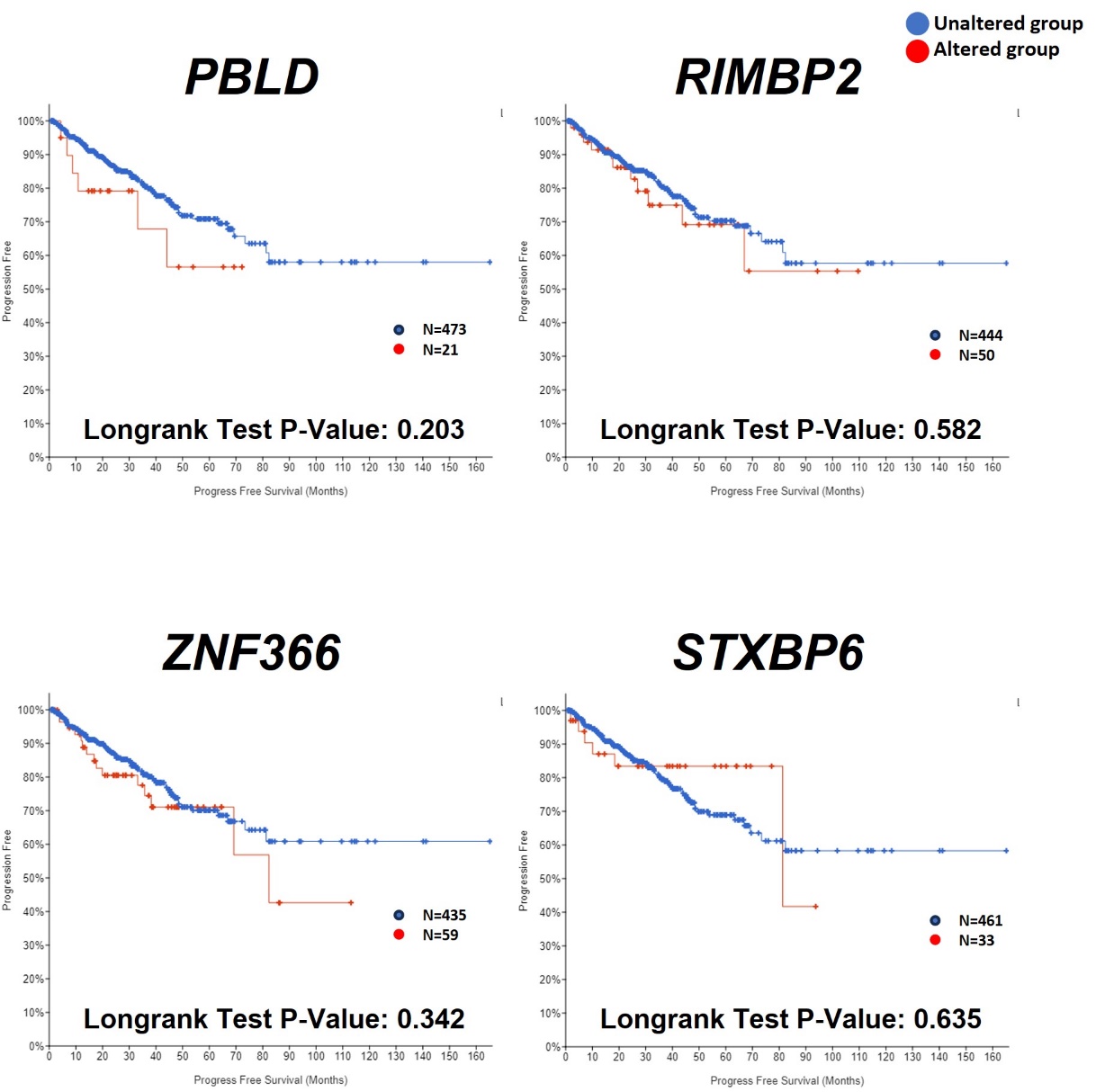

Supplement: Supplementary file 1 — Supplementary Information. [file 41598_2023_46068_MOESM1_ESM.docx]
